# Prostate specific membrane antigen (PSMA) and Prostate Cancer Staging: is our current conventional staging obsolete?

**DOI:** 10.1590/S1677-5538.IBJU.2020.0997

**Published:** 2021-01-20

**Authors:** Melissa Segura Céspedes, Jan Philipp Radtke, Xavier Cathelineau, Rafael Sanchez-Salas

**Affiliations:** 1 Klinikum Darmstadt Department of Urology Darmstadt Germany Department of Urology, Klinikum Darmstadt, Darmstadt, Germany;; 2 University Hospital Essen Department of Urology Essen Germany Department of Urology, University Hospital Essen, Essen, Germany;; 3 University Hospital Essen German Cancer Consortium Essen Germany German Cancer Consortium (DKTK)-University Hospital Essen, Essen, Germany;; 4 German Cancer Research Center Department of Radiology Heidelberg Germany Department of Radiology, German Cancer Research Center (DKFZ), Heidelberg, Germany;; 5 L`Institut Mutualiste Montsouris Department of Urology Paris France Department of Urology, L`Institut Mutualiste Montsouris, Paris, France

## INTRODUCTION

The extent of prostate cancer (PC) with imaging is crucial for therapeutic decision-making, particularly in patients suffering from high-risk localized PC or at risk of extended disease (1). In addition, adequate staging and tailored stratification might lead to a positive impact on the natural evolution of the disease, particularly nodal staging (1-5).

Despite careful and appropriate selection of patients before radical prostatectomy (RP) or external beam radiotherapy (EBRT), relapse following treatment with curative intent is common in approximately 30% of men (6, 7). One reason might be due to limitations of existing standard conventional imaging using computed tomography (CT) and bone scintigraphy, namely both low sensitivity and specificity to detect non-localized disease, particularly in detecting tumor-positive lymph nodes of regular size and metastatic burden in low PSA-levels (8-13). As the diagnostic capability of these conventional imaging modalities is limited, as CT has a sensitivity of only about 40% and bone scintigraphy a cumulative sensitivity of approximately 80%, there has been an unmet need for more advanced imaging modalities that better detect loco-regional and distant metastatic disease in order to guide the appropriate management of patients (9, 10 ,14). Multiparametric MRI (mpMRI) has gained widespread utilization prior to prostate biopsy to detect tumor foci within the prostate (15). In this context three Level I evidence trials have demonstrated superiority as compared to conventional prostate imaging (16-18). For local staging purposes, a meta-analysis on mpMRI showed a limited sensitivity of 57% for EPE-detection, but 90% specificity (19). This disappointing results are because MRI cannot detect microscopic extraprostatic extension and significantly underestimates tumor volume by approximately 30% (1, 19-22). In terms of nodal staging, diffusion-weighted imaging as part of the MR scan has shown promising results with an accuracy of about 83%, but generalizability is limited due to reader experience and different imaging techniques, sequences and MR scanners (23). Advanced MR imaging techniques, like whole-body MRI and Ultra-small superparamagnetic iron oxide (USPIO) enhanced MRI also demonstrate promising results, but availability is also limited (24-26).

Novel imaging might improve detection accuracy and subsequent outcomes by more accurately defining disease extent at the outset, enabling a more tailored multimodal treatment plan (27). One of these promising candidates is the mostly ^68^Gallium (Ga)- Prostate Specific Membrane Antigen (PSMA) positron emission tomography (PET)/computed tomography (PET/CT) (28-30). ^68^Ga-PSMA-PET/CT is a non-invasive diagnostic technique to image PC with increased (PSMA, glutamate carboxypeptidase II, EC 3.4.17.21) expression (28).

### Basically

PSMA is a transmembrane protein primarily present in all prostatic tissues (28, 31). However, increased PSMA expression is seen in a variety of malignancies, however, most notably in PC (31-33). Immunohistochemical studies have shown that PSMA expression increases in case of de-differentiated, more aggressive, metastatic, and also in castration-resistant disease and its expression level is a significant prognosticator for disease outcome (28, 31, 33).

Therefore, this tool represents a symbiosis in the evaluation between tumor microenvironment and imaging, and it should be able to provide a more refined prostate cancer stratification (20).

In 2020, multiple types of radiopharmaceutical tracers, including various PSMA-tracers are available. The most frequently deployed according to their specificity in PC, are Fluorine 18 (^18^F)- and Gallium 68 (^68^Ga)-labeled PSMA (34). Until know, ^68^Ga-PSMA-PET/CT has demonstrated these high rates of specificity with increased levels of sensitivity as compared to conventional imaging in both staging of primary tumor and in biochemical recurrence (29, 35). However, the sensitivity strongly depends on PSA-levels, with low sensitivity rates in PSA-levels <0.2ng/mL and higher rates >1.0ng/mL, and lymph node and tumor diameter (36-38). In addition, the PSMA-targeted ^18^F-DCFPyL (2-(3-{1-carboxy-5-[(6-18F-fluoro-pyridine-3-carbonyl)-amino]-pentyl}-ureido) pentanedioic acid) is a novel and promising tracer, demonstrating both, improved positive and negative predictive value, as compared to standard imaging in the recently published OSPREY trial (39).

In this context, the recently published proPSMA trial by Hofman et al. should be further elucidated (27). This study is of particular interest due to several reasons. The study design comprised 302 men with high-risk PC, that where prospectively randomized in a multicentric fashion to either conventional imaging (CT and bone scintigraphy) or ^68^Ga-PSMA-PET/CT as first imaging modality.

The primary aim was to determine the accuracy of staging between ^68^Ga- PSMA-PET/ CT and conventional imaging. Importantly, men underwent the opposite imaging modality after the first-line imaging prior to treatment with RP or radiotherapy (27).

Hofman et al. found that PSMA PET/CT had a significant higher accuracy of 27% (92% versus 65%, p <0.001) as compared to conventional imaging. Also both sensitivity (38% vs. 85%) and specificity (91% vs. 98%) were lower for conventional imaging (27). Subgroup analyses also showed superiority in patients with pelvic nodal metastases and a 22% absolute difference for distant metastases.

Of great importance was that conventional imaging conferred management change with a high or medium effect, defined as a change in management intent or modality, or change in modality delivery in 23 men (15%, 95% confidence interval (CI) 10-22), compared with 41 men (28%, CI 21-36) who underwent first-line PSMA PET-CT (p=0.008). In detail, 20 (14%) of 148 patients were directed from curative to palliative-intent treatment after first- line PSMA PET-CT, 11 (7%) had a change in radiotherapy technique, and 11 (7%) in surgical technique (27).

First line conventional imaging conferred management changes less frequently (15% vs. 28%) and yielded more equivocal findings (23% vs. 7%). For those who underwent a second line imaging, management change occurred in 5% in conventional imaging vs. 27% in PSMA PET/CT.

In addition, PSMA PET/CT was not only associated with a lower level of radiation exposure of 8.4mSv as compared to 19.3mSv (p <0.001), but also did not lead to any adverse events.

In conclusion proPSMA delivers evidence from a prospective randomized trial that 68Ga-PSMA-PET-CT is in favor of applied dose, sensitivity, specificity, less equivocal imaging findings and improved management effect as compared to conventional imaging using abdominal cross-sectional imaging and bone scintigraphy.

Yet, some important factors need to be discussed: Although patients underwent selective cross-over to assess utility for second- line imaging, the primary endpoint was head-to-head comparison of first-line imaging before cross-over (27). Limitations though include that analysis of the second-line imaging was of a subset of patients and not a randomized comparison (27). In addition, the authors mentioned that although potential confounders were reduced by randomization, the inability to blind the imaging modality introduced potential bias (27). Thirdly, reflecting real-world practice, histopathologic assessment was not feasible in all participants, especially those with pelvic nodal metastases who underwent radiotherapy. To overcome issues regarding pathology standard, the study design included follow-up with repeat imaging six months after therapy initiation (27).

One of the most important acknowledgments of the study is, that although initial PSMA PET-CT led to a significant higher rate of changes in intended management, the cross-over design limited the ability to identify specific improvements of patient outcomes between the imaging modality groups in longer term follow-up. In particular, effects on progression free survival (PFS), changes in systematic treatments, like delay of androgen deprivation therapy (ADT) or more sophisticated overall survival (OS) cannot determined using a cross-over design. However, it has to be acknowledged that the study design focused on the comparative accuracy of PSMA PET-CT compared with conventional imaging and has inherent benefits in terms of diagnostic accuracy and safety for patients.

In this context, it will be interesting to see if improving diagnostic accuracy, that can lead to prevent futile attempts at cure or better direct locoregional therapies, can be translated into improved long-term benefits in this setting. Furthermore, earlier detection of systemic metastases could also be beneficial for patients because the efficacy of therapies is greater when the burden of disease is low (40). However, this was not an endpoint of the proPSMA study.

Other authors like Yaxley et al. have contributed as well on this topic with a retrospective review in 1253 men using ^68^Ga-PSMA PET/CT for initial staging (41). The primary outcome was to determine the risk of metastasis based on Gallium ^68^PSMA PET/CT as well with histological biopsy International Society of Urological Pathology (ISUP) grade, prostate-specific antigen level, and staging with pre-biopsy multiparametric magnetic resonance imaging (mpMRI) (41).

Their results also support the use of Gallium ^68^PSMA PET/CT for primary staging of prostate cancer metastatic disease in 12.1% of men, including 8.2% with a PSA level of <10ng/mL and 43% with a PSA level of >20ng/mL (41).

Current European guidelines state a growing evidence on the performance of ^68^Ga-PSMA PET/CT in initial staging (1). Perera et al. contributed to this topic with a recent systematic review including 37 studies and comprising a total of 4790 patients (29). They found that about 90% of high-risk patients on primary-staging were PSMA-PET positive (29). Luiting et al. published a systematic review comprising 11 studies, demonstrating a variable per-patient sensitivity between 33% and 100% and per-patient specificity of 80-100% to detect lymph node metastases using RP and extended lymph node dissection as reference standard (35). Per-node sensitivity was analogous variable with 24-96% and per-node specificity very high with 98-100% (35).

In this context, EAU Guidelines concluded that the field of non-invasive nodal and metastatic staging of PC is evolving very rapidly (1). Evidence shows that choline PET/CT, MRI and PSMA PET/CT provide a more sensitive detection of LN and bone metastases than the classical work-up associating bone scan and abdominopelvic CT (27, 42-44). It could then be tempting to conclude that bone scan and abdominopelvic CT must be replaced by more sensitive tests in all patients undergoing initial PCa staging (1).

Recent NCCN guidelines considered the performance of an initial stratification and staging for men suffering from at least intermediate-risk disease with a bone imaging including plain films like CT and MRI (45). Those imaging modalities could be accompanied by ^18^F sodium fluoride PET/CT or PET/MRI, C-11 choline PET/CT or PET/MRI for equivocal results on initial bone scan (45). However, information on PSMA-PET imaging are lacking (45).

Beyond the potential benefits of this imaging tool, PSMA assessment is not without limitations.

First, the spectrum of benign and malignant non-prostatic conditions with high PSMA-radiotracer uptake may be misguided for sites of PC as a potential false positive. To mention some of them, we can see an increased uptake of ^68^Ga-PSMA -11 or ^18^F-DCFPyL in ganglia of the sympathetic trunk along the vertebra, which can be mistaken with bone metastasis (46). This is a common phenomenon in approximately 50.90% of cases (46). However, PSMA-avidity is mostly teardrop- or nodular-shapen in lymph node metastases in 50-70% of cases and only rarely (about 1%) in sympathetic trunk uptake (46). Also in benign bone pathologies, where there is a setting of increased vascularity, bone remodeling, and reparative processes like in Paget Syndrome or anemia (47).

Secondly, on the other hand, PC with neuroendocrine differentiation (NEPC) has been increasingly reported as a common cause of false negative PSMA-targeted PET/CT (15). However, rates of NEPC are varying between x and y percent (48). In this context, a third limitation is that about 5-10% of PCs are PSMA negative, so potential metastases are not avid due to missing tracer uptake (49).

Fourthly, PSMA-PET imaging is not available in most countries outside north, middle and southern Europe, as well as Australia, Asia and the US. As both cost- and time-consumption are still challenging, widespread implementation is limited. However, we acknowledge that this is also the case for alternative modern staging tools, like whole-body MRI and DCFPyL-PSMA-PET.

As mentioned before, further randomized control trials are needed to identify the impact on the outcome and probably OS after a change in patient management based on new evidence provided by PSMA PET/CT, and how this would lead to more accurate and successful disease-control. One example will be the upcoming multicentric PRIMARY trial (50).

The primary outcome of this study, that transfers PSMA-PET imaging to the screening setting, is to determine the additive value of ^68^Ga-PSMA-PET/CT when combined with mpMRI detecting clinically significant PC (csPC) in men undergoing initial biopsy for suspicion of PC, and to determine the proportion of men who could have avoided prostate biopsy with positive mpMRI (PI-RADS ≥3) but negative PSMA-PET/CT (50). The PRIMARY trial will be a multicenter, prospective, cross-sectional study that meets the criteria for level 1 evidence in diagnostic test evaluation (50). PRIMARY will also investigate if a limited (pelvic-only) PSMA-PET/CT in combination with routine mpMRI can reliably discriminate men with csPCa from those without csPCa, using transperineal template+targeted (PSMA-PET/CT and/or mpMRI) biopsies as reference test (50).

In conclusion, PSMA-PET/CT has proved so far to be a highly specific imaging modality in staging of PC with higher sensitivity rates as compared to standard imaging methods (27). In addition, it has the potential to change patient’s management. This has also be proven in the recent published proPSMA study (27). While data on the impact of applying PSMA-PET/CT as first-line staging in PC on long-term outcomes and AS are lacking and staging accuracy depends on PSA-levels and tumor- and lymph node-size, recent guidelines focus on the high potential of this imaging tool, potentially changing guidelines (1, 37, 38). Data on PSMA-PET with novel tracers and comparisons to whole-body MRI are eagerly expected, as availability of PSMA-PET/CT is still limited.
